# Clinical significance of site-specific metastases in pancreatic cancer: a study based on both clinical trial and real-world data

**DOI:** 10.7150/jca.50317

**Published:** 2021-01-18

**Authors:** Lixia Wu, Lina Zhu, Kequn Xu, Siyuan Zhou, Yang Zhou, Tiening Zhang, Junjie Hang, Benny Chung-Ying Zee

**Affiliations:** 1 Department of Oncology, Shanghai JingAn District ZhaBei Central Hospital, Shanghai 200070, China.; 2 Changzhou No. 2 People's Hospital, Affiliated Hospital of Nanjing Medical University, Changzhou 213000, China.; 3 Department of Radiotherapy, Shanghai General Hospital, Shanghai 200080, China.; 4 JC School of Public Health and Primary Care, The Chinese University of Hong Kong, Prince of Wales Hospital, Shatin 999077, Hong Kong, China.

**Keywords:** lung metastasis, pancreatic cancer, predictive value, prognosis, site-specific metastases

## Abstract

**Background:** There is limited consensus on whether metastatic patterns are correlated with prognosis and treatment efficacy in pancreatic cancer. A better understanding of clinical implication of the metastatic patterns is pivotal for therapeutic decision-making and drug development.

**Methods:** This study included 977 patients with metastatic pancreatic cancer (MPC) in three cohorts. The training cohort included 273 patients from clinical trial NCT00574275 and 367 patients from clinical trial NCT01124786. As the validation cohort, 337 patients from Changzhou No.2 People's Hospital and Shanghai General Hospital were enrolled. The correlations between different patterns of metastases and clinicopathological characteristics were investigated with the Pearson Chi-Square test. Kaplan-Meier analysis and log-rank test were applied to analyze the survival outcomes among groups with different metastatic patterns. The prognostic value of the number of metastatic sites and other variables was evaluated using the Cox regression model.

**Results:** MPC patients aged ≥65 years had a higher rate of lung metastasis and those with liver metastasis were prone to have a high level of carbohydrate antigen 19-9 (CA19-9). Additionally, patients with isolated lung metastasis had much better overall survival (OS) than those with isolated liver or peritoneum metastasis. Cox regression analyses showed that the number of metastatic sites was an independent prognostic factor for OS in patients with MPC. Furthermore, for patients with one-site or two-site metastasis, there was a significant difference in OS among patients receiving no chemotherapy, monotherapy and combination therapy. However, for patients with more than two metastatic sites, receiving combination therapy or monotherapy showed limited superiority in OS over receiving no chemotherapy.

**Conclusion:** MPC patients with isolated lung metastasis had better OS than those with isolated liver or peritoneum metastasis. Moreover, the number of metastatic sites showed prognostic and predictive value in patients with MPC.

## Introduction

Pancreatic cancer is the seventh leading cause of cancer-related mortality worldwide in both men and women, with an overall five-year survival of less than 6% 1. Approximately 80% of patients with pancreatic cancer are at an advanced stage when they are initially diagnosed 2. Although marked progress has been made in the treatment of pancreatic cancer, chemotherapy remains the primary treatment for patients with metastatic disease. However, only a small proportion of patients benefit from targeted therapy and immunotherapy 3,4.

Liver is the most common site of distant metastases in pancreatic cancer, but the cancer cells can also metastasize to other distant organs 5. The specific metastatic sites may reflect the molecular background and clinicopathological characteristics of pancreatic cancer subtypes 6-9. For example, Oweira et al. showed that pancreatic cancer patients with isolated liver metastases had worse survival outcome compared with those with isolated lung or distant nodal metastases 10. According to the eighth edition of the pancreatic cancer staging published by the American Joint Committee on Cancer (AJCC), pancreatic cancer with distant metastases are defined as M1 11. Additionally, only a limited number of studies have investigated the occurrence rate and prognostic value of different metastatic patterns in pancreatic cancer 12-14. Furthermore, almost no guidelines have taken the number and site of metastases into consideration for the treatment of metastatic pancreatic cancer (MPC).

There has been limited consensus on whether metastatic patterns are correlated with different prognosis and treatment efficacy in pancreatic cancer 15. However, a better understanding of the metastatic patterns is pivotal for therapeutic decision-making and drug development. In this study, we addressed this issue with data from clinical trials and real-world study.

## Methods

### Patients

A total of 273 patients with MPC in clinical trial NCT00574275 and 367 patients with MPC in clinical trial NCT01124786 with complete records of clinicopathological features were selected from the Project Data Sphere (PDS), a not-for-profit initiative allowing collective historical cancer clinical trial data to be shared in public 16. NCT00574275 was a multinational, double-blind, phase III trial that assessed the efficacy of aflibercept in patients with MPC treated with gemcitabine. All 273 patients were in the comparator arm and were treated with gemcitabine and placebo 17. NCT01124786 was a multicenter, randomized, phase II trial that compared CO-101 with gemcitabine as first-line therapy in patients with MPC 18. Among the 367 patients, 182 patients received CO-101 alone and 185 patients received gemcitabine alone. All patients from the two clinical trials were enrolled in the training cohort. Additionally, 337 patients with MPC from Changzhou No. 2 People's Hospital and Shanghai General Hospital were enrolled in the validation cohort. The following inclusion criteria were applied: (1) pathologically confirmed pancreatic adenocarcinoma; (2) no concurrent cancer at another site; and (3) complete records of clinicopathological features. Ethical approval was obtained by the ethics committees of Changzhou No. 2 People's Hospital.

### Statistical analysis

The Venn plot showing the distribution of patients with different patterns of metastases was generated with the online tool Bioinformatics & Evolutionary Genomics (http://bioinformatics.psb.ugent.be/webtools/Venn/). Statistical analyses were performed with SPSS statistical software (version 21.0, SPSS Inc., Chicago, IL, USA). Descriptive statistics are presented as median level and 95% confidence interval (95%CI). To assess the correlations between the metastatic sites and clinicopathological characteristics, patients were classified into two groups according to gender (male or female), age (<65 or ≥65), Eastern Cooperative Oncology Group performance status (ECOG PS) (0-1 or ≥2), body mass index (BMI) (normal weight or others), primary tumor location (head and neck or body and tail), and the level of CA19-9 (<1000 or ≥1000). Comparison between groups was conducted using the Pearson Chi-Square test. Kaplan-Meier analysis and log-rank test were used to evaluate the survival outcome between different groups. The prognostic value of the number of metastatic sites and variables was evaluated by Cox regression model. For each factor, we calculated the hazard ratio (HR) and corresponding 95%CI. Two-sided P<0.05 was considered statistically significant.

## Results

### Patient characteristics

**Figure 1:**
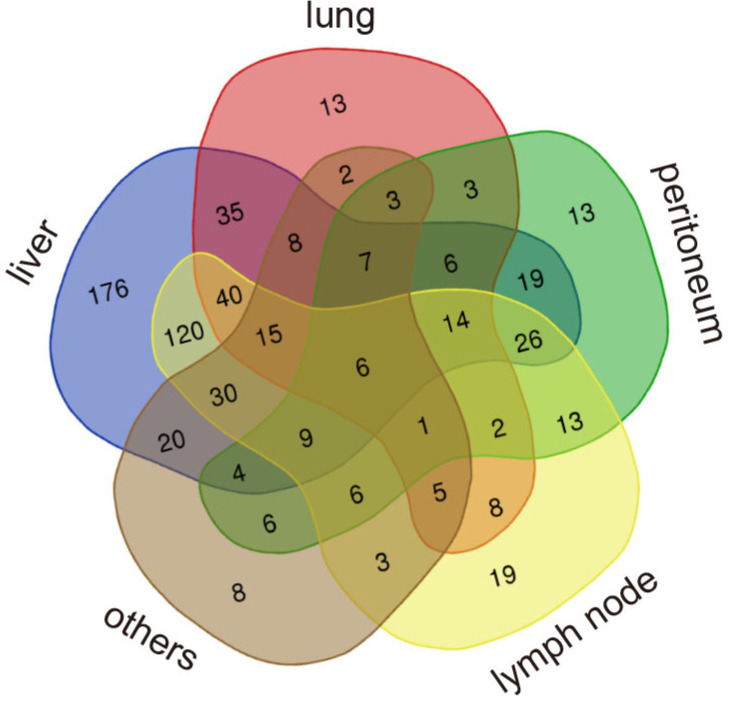
The distribution of patients with different patterns of metastases.

The baseline clinicopathological characteristics of patients with MPC in both training cohort (n=640) and validation cohort (n=337) are listed in Table 1. In the training cohort, most patients had a good performance status of 0 to 1 (n=623, 97.3%) and 92.5% were white. Additionally, all patients received gemcitabine or its conjugate CO-101. In the validation cohort, all the patients were Asian and only half had a good performance status of 0 to 1 (n=167, 49.5%). In the total validation cohort, 17.2% of patients received no chemotherapy, 49.9% received monotherapy and 32.9% received combination therapy. Liver was the most common metastatic site in both cohorts, with a rate of 83.6% (535/640) in the training cohort and 71.2% (240/337) in the validation cohort. The metastatic rate to lung was similar in the training cohort (26.3%) and validation cohort (26.7%). A small portion of patients also had kidney or adrenal metastases in the training cohort (n=21, 3.3%) and validation cohort (n=33, 9.8%). Data on other less common metastatic sites including spleen, bone, soft tissue and brain, as well as the number of metastatic sites in the training cohort and validation cohort, are presented in Supplementary Figure 1. The distribution of patients with different patterns of metastases in the training cohort is shown in Venn plots (Figure 1).

### Correlations between clinicopathological characteristics and metastatic sites

In the training cohort, patients aged <65 years were more prone to have liver metastases (P=0.009) while patients aged ≥65 years had a higher metastatic rate of lung (P=0.027) (Table 2). Additionally, patients with abnormal BMI more frequently showed lung metastasis (P=0.040). Moreover, patients with the primary tumor location in body and tail had higher rate of peritoneum metastasis (P=0.023). Intriguingly, CA19-9 ≥1000 U/ml was significantly correlated with liver metastases (P=0.001). Supplementary Table 1 showed that patients aged ≥65 also had a higher metastatic rate in lung (P=0.043) and patients with liver metastasis were more prone to have high levels of CA19-9 (P=0.013) in the validation cohort. Nevertheless, no difference in other characteristics was observed between patients with or without metastases in liver, lung or peritoneum.

### Survival outcome among liver, lung and peritoneum metastasis groups

**Figure 2:**
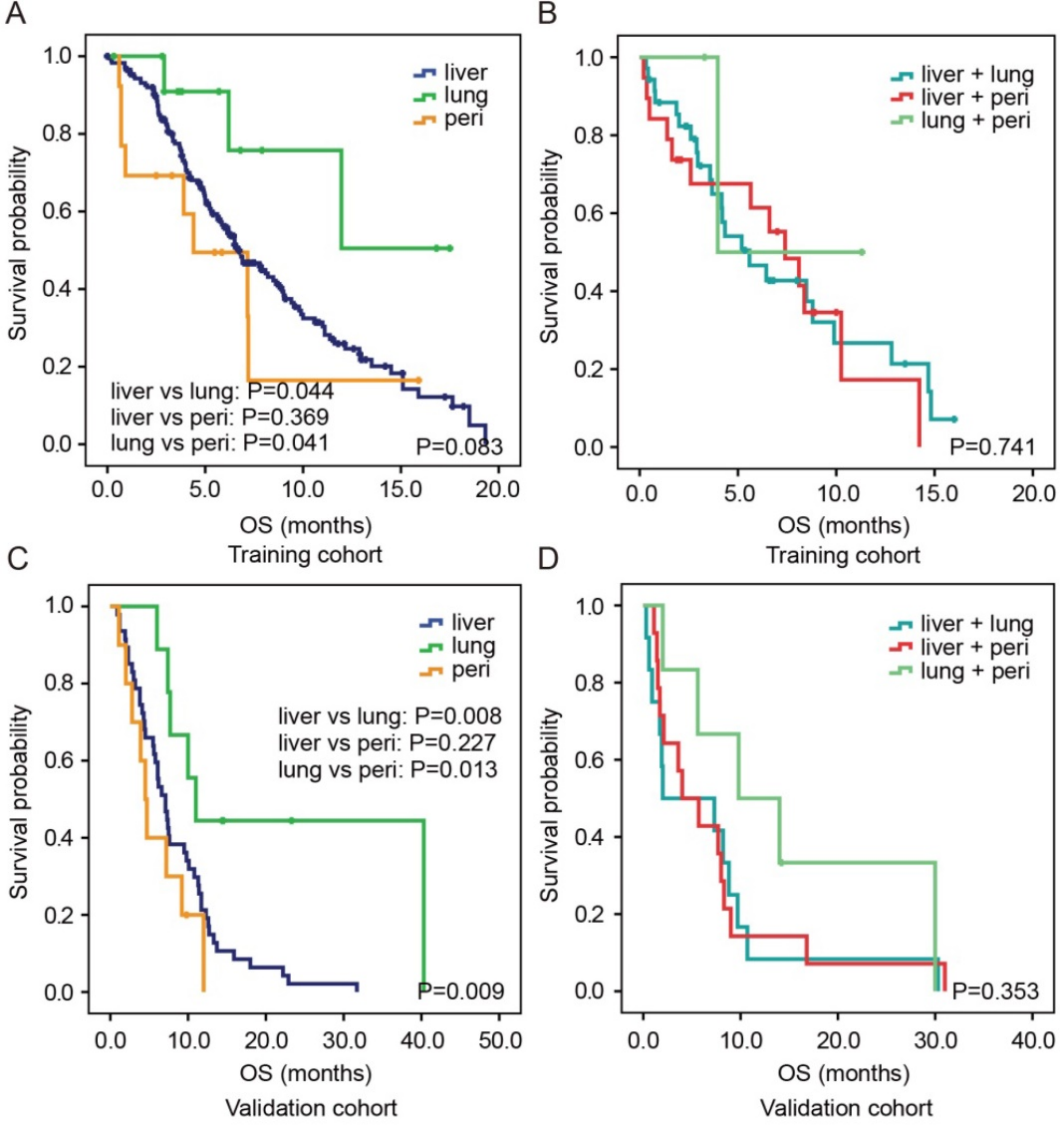
Kaplan-Meier estimates OS according to different metastatic patterns in both the training cohort (A, B) and validation cohort (C,D). Abbreviation: peri, peritoneum.

In the training cohort, the median OS of patients with isolated lung metastasis was significantly longer than that of patients with isolated liver metastasis (P=0.044) or peritoneum metastasis (P=0.041, Figure 2A). However, no significant difference was identified in OS between patients with isolated liver metastasis and those with peritoneum metastasis (P=0.369). For two-site metastases, there were also no significant differences in OS among patients with liver and lung, liver and peritoneum, and lung and peritoneum metastases (all P>0.05, Figure 2B). Similar results were observed in the validation cohort (Figure 2C and 2D).

### Prognostic value of the number of metastatic sites

**Figure 3:**
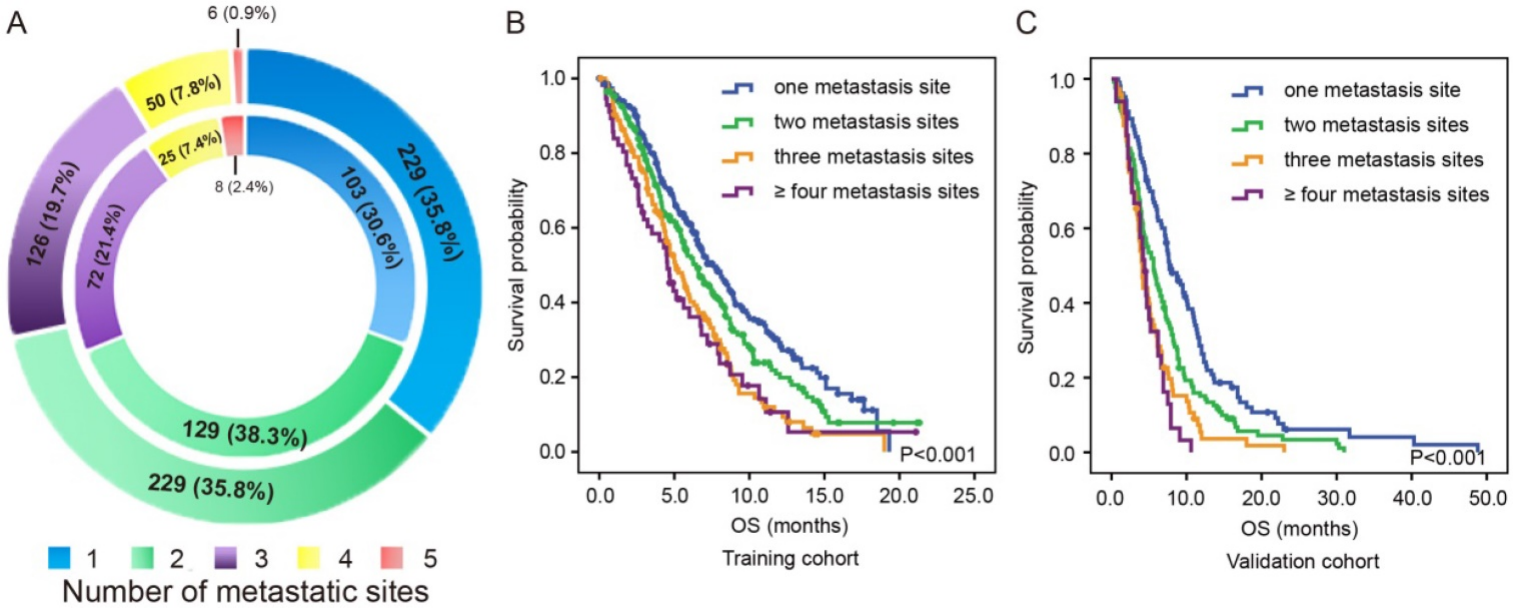
The distribution (A) and survival outcome according to the number of metastatic sites in both the training cohort (B) and validation cohort (C).

As shown in the outer ring of Figure 3A, there were 229 (35.8%) patients with isolated metastases, 229 (35.8%) patients with two-site metastases, 126 (19.7%) patients with three-site metastases, 50 (7.8%) patients with four-site metastases and 6 (0.9%) patients with five-site metastases in the training cohort. As shown in the inner ring of Figure 3A, there were 103 (30.6%) patients with isolated metastases, 129 (38.3%) patients with two-site metastases, 72 (21.4%) patients with three-site metastases, 25 (7.4%) patients with four-site metastases and 8 (2.4%) patients with five-site metastases in the validation cohort.

In the training cohort, patients with two-site metastases showed the tendency of having poorer OS than patients with one metastatic site (6.4 months vs. 7.6 months, P=0.058) but exhibited better OS than those with three metastatic sites (6.4 months vs. 5.1 months, P=0.010) and those with more than three metastatic sites (6.4 months vs. 4.5 months, P=0.013). However, no significant difference was observed between the OS of patients with three metastatic sites and those with more than three metastatic sites (5.1 months vs. 4.5 months, P=0.587, Figure 3B). Similar results were observed in the validation cohort (Figure 3C). Patients with two-site metastases had poorer OS than those with one metastatic site (5.6 months vs 7.9 months, P=0.001) but had better OS than those with three-site metastases (5.6 months vs 4.1 months, P=0.023) and those with more than three metastatic sites (5.6 months vs 4.4 months, P=0.006). Likewise, there was no significant difference in OS between patients with three metastatic sites and those with more than three metastatic sites (4.1 months vs 4.4 months, P=0.392).

In univariate analysis, ECOG PS, CA19-9 and the number of metastatic sites were all significantly correlated with OS. All of these factors were subsequently analyzed in multivariate analysis, and all three factors showed independent prognostic value in the training cohort (Table 3). Because of the heterogeneity in outcomes of chemotherapy for patients in the validation cohort, we included chemotherapy into analysis by classifying patients into three groups: no chemotherapy, monotherapy and combination therapy. In univariate analysis, ECOG PS, CA19-9, number of metastatic sites and chemotherapy were significantly correlated with OS. However, only the number of metastatic sites and chemotherapy showed independent prognostic value in multivariate analysis (Supplementary Table 2).

### The predictive value of metastatic sites in palliative treatment

**Figure 4:**
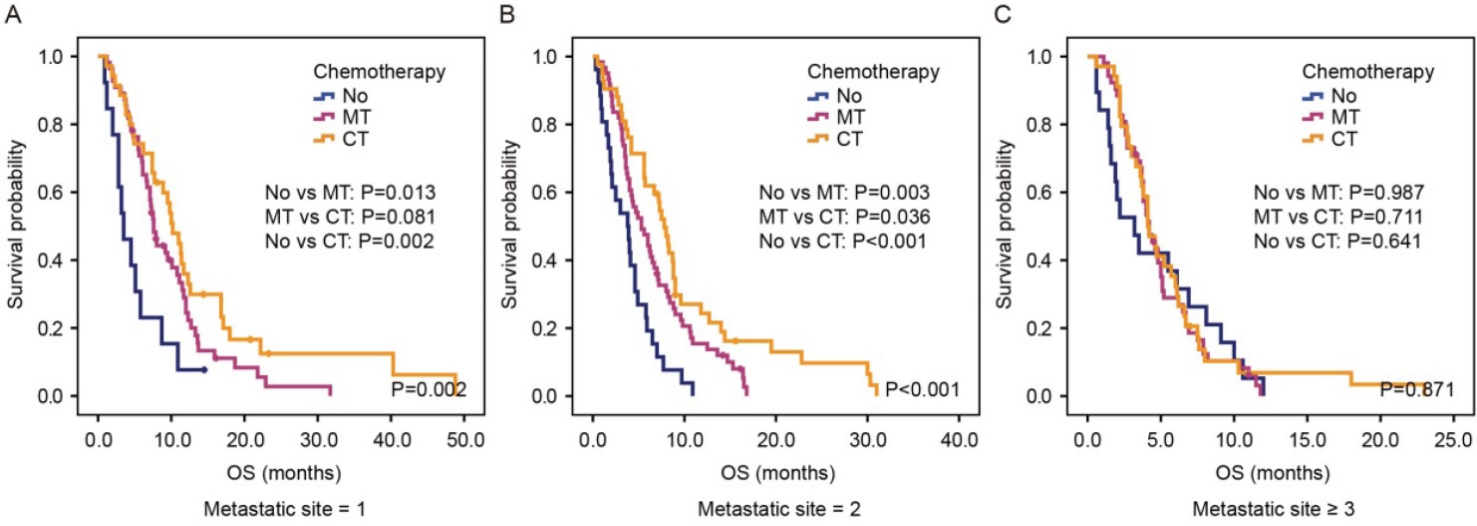
Kaplan-Meier estimates OS according to chemotherapy in the group of patients with one metastatic site (A), two metastatic sites (B) and more than two metastatic sites (C) in the validation cohort. Abbreviation: No, no chemotherapy; MT, monotherapy; CT, combination therapy.

We next investigated whether the number of metastatic sites affected the efficacy of chemotherapy by dividing patients into three groups: patients with one-site metastasis, patients with two-site metastasis and patients with more than two metastatic sites. In the first group, patients receiving monotherapy or combination therapy showed better OS than those receiving no chemotherapy (P=0.002, Figure 4A). Furthermore, a trend was seen that combination therapy was superior compared with monotherapy in this group of patients (P=0.081). Likewise, for patients with two-site metastasis, there was a significant difference among those receiving no chemotherapy, monotherapy and combination therapy (3.8 months vs. 5.3 months vs. 7.8 months, P<0.001, Figure 4B). However, for patients with more than two metastatic sites, patients receiving combination therapy or monotherapy showed no superiority in OS than patients receiving no chemotherapy (4.1 months vs. 4.2 months vs. 3.2 months, P=0.871, Figure 4C).

## Discussion

The survival rate of MPC is very dismal, but few studies have examined the survival rate and treatment effect in view of the number and sites of metastases. The main findings of this study were the following: (1) patients aged ≥65 years had a higher metastatic rate of lung than those aged <65 years and patients with liver metastasis were prone to have high level of CA19-9; (2) patients with isolated lung metastasis had much better OS than those with isolated liver or peritoneum metastasis; (3) the number of metastatic sites was an independent prognostic factor for OS in patients with MPC and (4) for patients with one-site or two-site metastasis, there was a significant difference in OS among those receiving no chemotherapy, monotherapy and combination therapy, while for patients with more than two metastatic sites, combination therapy or monotherapy showed no superiority in OS over best support care.

Pancreatic cancer can metastasize to different organs by lymphogenic, hematogenous and perineural route or by direct spread 19. In a large-scale autopsy study in pancreatic cancer, liver was the most common metastatic site, followed by distant lymph node, lung and peritoneum. Other less common metastatic sites include kidney, adrenal, bone, spleen, gallbladder, omentum and, brain 5. Our work demonstrated a similar distribution of metastatic pattern, as shown in Table 1.

Intriguingly, we found that patients with liver metastasis were prone to have a high level of CA19-9. Because CA19-9 is usually considered as a measure of pancreatic tumor burden, it is reasonable that patients with higher tumor burden will have an increased chance to develop liver metastasis. The level of CA19-9 is also determined by Lewis antigens and associated genes like α1-2 fucosyltransferase (FUT2) and α1-4 fucosyltransferase (FUT3) genes 20,21. However, there is still no evidence to suggest that the expression of these genes is correlated with liver metastasis.

In a case series, Deeb et al. observed a longer than expected survival in five pancreatic cancer patients with pulmonary metastases 22. Katz et al. found that the most common metastatic site was the lung among long-term survivors with pancreatic cancer 23. More recently, Lovecek et al. found that pancreatic adenocarcinoma patients with metachronous pulmonary metastases had a better OS of 31.81 months 6. In our work, patients with isolated lung metastasis also had much better OS than those with isolated liver or peritoneal metastasis in both the training cohort and validation cohort. Emerging evidence has suggested that surgery should be considered for patients with isolated lung oligometastases 6,7,19,24. However, our work demonstrated that patients with age ≥65 had a much higher metastatic rate of lung than those with age <65. As old age usually means higher risk and more difficulty in recovering from interventions like surgery, individual evaluation should be carefully conducted for these patients.

Previous studies showed that the numbers of distant metastases were significantly correlated with outcomes in some types of cancer 25,26, but a similar correlation was not seen in pancreatic cancer. Oweira et al. found no significant difference in OS between pancreatic cancer patients with single metastasis and multiple metastases (P=0.409) 10. Intriguingly, in our work, the number of metastatic sites was an independent prognostic factor for OS in patients with MPC. These findings may suggest that the number of metastatic sites plays a more important role than the number of distant metastases in patient' outcome. Moreover, with the data from the real-world study, we found that for patients with one or two-site metastasis, the OS of patients receiving combination therapy was much longer than those receiving monotherapy or no chemotherapy. However, for patients with more than two metastatic sites, receiving combination therapy or monotherapy showed no superiority in OS than receiving no chemotherapy. Because of the small sample size and the retrospective design of this study, more studies are warranted to investigate whether other interventions, like chemotherapy, are necessary for MPC patients with more than two metastatic sites.

This study has several limitations. First, there was an evident heterogeneity in the data source and race of patients in the training cohort and validation cohort. Second, the relatively small sample size for every metastatic pattern was likely to make the results less robust. Third, other factors, such as primary tumor size, living habit and comorbidities, were not included in the analysis, which may lead to unexpected bias. Additionally, we did not take the number of metastases in each metastatic site into consideration because of the limitation of the data source. Thus, further research is needed to verify and expand on the clinical significance of site-specific metastases in pancreatic cancer.

In conclusion, MPC patients with isolated lung metastasis had better OS than those with isolated liver or peritoneum metastasis. Additionally, the number of metastatic sites showed both prognostic and predictive value in patients with MPC.

## Supplementary Material

Supplementary figures and tables.

## Figures and Tables

**Table 1:** Baseline clinicopathological characteristics of patients with MPC

	Training cohort 1 (n=273)	Training cohort 2 (n=367)	Validation cohort (n=337)
**Age (years)**	61 (34-84)	62 (26-86)	63 (27-89)
Gender			
Male	156 (57.1%)	219 (59.7%)	208 (61.7%)
Female	117 (42.9%)	148 (40.3%)	129 (38.3%)
**Performance status**			
ECOG PS=0	98 (35.9%)	76 (20.7%)	23 (6.8%)
ECOG PS=1	159 (58.2%)	290 (79.0%)	144 (42.7%)
ECOG PS=2	16 (5.9%)	1 (0.3%)	151 (44.8%)
ECOG PS=3	0	0	19 (5.6%)
**Body mass index**			
Underweight (<18.5)	16 (5.9%)	24 (6.5%)	65 (19.3%)
Normal weight (18.5-25)	146 (53.5%)	199 (54.2%)	240 (71.2%)
Overweight (25-30)	80 (29.3%)	98 (26.7%)	31 (9.2%)
Obese (≥30)	31 (11.4%)	36 (9.8%)	1 (0.3%)
Missing	0	10 (2.7%)	0
**Race**			
Asian	0	5 (1.4%)	337 (100%)
White	265 (97.1%)	327 (89.1%)	0
Black	0	3 (0.8%)	0
Others	8 (2.9%)	13 (3.5%)	0
Missing	0	19 (5.2%)	0
**Primary tumor location**			
Head and neck	115 (42.1%)	0	146 (43.3%)
Body and tail	86 (31.5%)	0	190 (56.4%)
Entire pancreas	72 (26.4%)	0	1 (0.3%)
Unknown	0	367 (100%)	0
**Metastatic site**			
Liver	214 (78.4%)	321 (87.5%)	240 (71.2%)
Lung	67 (24.5%)	101 (27.5%)	90 (26.7%)
Peritoneum	64 (23.4%)	74 (20.2%)	100 (29.7%)
Kidney or adrenal	15 (5.5%)	6 (1.6%)	33 (9.8%)
Lymph nodes	124 (45.4%)	193 (52.6%)	172 (51.0%)
Others	24 (8.7%)	92 (25.1%)	70 (20.8%)
**Chemotherapy**			
No	0	0	58 (17.2%)
Monotherapy	273 (100%)	367 (100%)	168 (49.9%)
Combination therapy	0	0	111 (32.9%)
CA 19-9 (U/mL)	1081.0(0.6-1743408.0)	2525.0 (0-2060000.0)	453.5 (0-3500.0)
Missing	14 (5.1%)	33 (9.0%)	5 (1.5%)

**Table 2:** Clinicopathological characteristics according to metastatic sites in the training cohort

Characteristics	Liver metastasis		*P*	Lung metastasis		*P*	Peritoneum metastasis		*P*
	Yes	No		Yes	No		Yes	No	
**Gender**									
Male	317	58	0.445	92	283	0.240	77	298	0.451
Female	218	47		76	189		61	204	
**Age**									
<65	343	53	0.009	92	304	0.027	83	313	0.637
≥65	192	52		76	168		55	189	
**ECOG PS**									
0-1	522	101	0.502	134	489	0.770	160	463	0.088
≥2	13	4		4	13		8	9	
**Body mass index**									
Normal weight	290	55	0.761	78	267	0.040	79	266	0.379
Others	237	48		85	200		57	228	
**Tumor location**									
Head and neck	87	28	0.108	25	90	0.952	16	99	0.023
Body and tail	73	13		67	17		23	63	
**CA19-9 (U/ml)**									
<1000	202	54	0.001	67	189	0.922	54	202	0.800
≥1000	299	38		87	250		74	263	

**Table 3:** Univariate and multivariate analysis of prognostic factors for OS in the training cohort

Characteristics	Univariate analysis			Multivariate analysis		
	HR	95%CI	*P*	HR	95%CI	*P*
**Gender**						
Male	Ref					
Female	0.998	0.907-1.097	0.965			
**Age**						
<65	Ref					
≥65	1.072	0.886-1.298	0.474			
**ECOG PS**						
0	Ref			Ref		
1	1.702	1.361-2.128	<0.001	1.617	1.279-2.044	<0.001
2	2.075	1.139-3.781	0.017	1.650	0.874-3.115	0.122
**Body mass index**						
Normal weight	Ref					
Others	1.007	0.833-1.218	0.939			
**Primary tumor location**						
Head and neck	Ref					
Body and tail	1.195	0.819-1.744	0.356			
**CA19-9 (U/ml)**						
<1000	Ref			Ref		
≥1000	1.638	1.337-2.005	<0.001	1.557	1.268-1.913	<0.001
**Number of metastatic sites**						
One	Ref			Ref		
Two	1.246	0.991-1.568	0.060	1.230	0.966-1.565	0.093
Three	1.734	1.339-2.246	<0.001	1.734	1.329-2.263	<0.001
More than three	1.887	1.342-2.653	<0.001	1.814	1.266-2.600	0.001

## Abbreviations

MPC: metastatic pancreatic cancer
CA19-9: carbohydrate antigen 19-9
OS: overall survival
AJCC: American Joint Committee on Cancer
PDS: Project Data Sphere
95%CI: 95% confidence interval
ECOG PS: Eastern Cooperative Oncology Group performance status
BMI: body mass index
HR: hazard ratio
FUT2: α1-2 fucosyltransferase
FUT3: α1-4 fucosyltransferase
