# Supplementary material for: Clinical significance of site-specific metastases in pancreatic cancer: a study based on both clinical trial and real-world data
Source: J Cancer. 2021 Jan 18;12(6):1715–21. doi: 10.7150/jca.50317 (PMC7890328; doi:10.7150/jca.50317)
Supplement: Supplementary file 1 — Supplementary figures and tables. [file jcav12p1715s1.pdf]

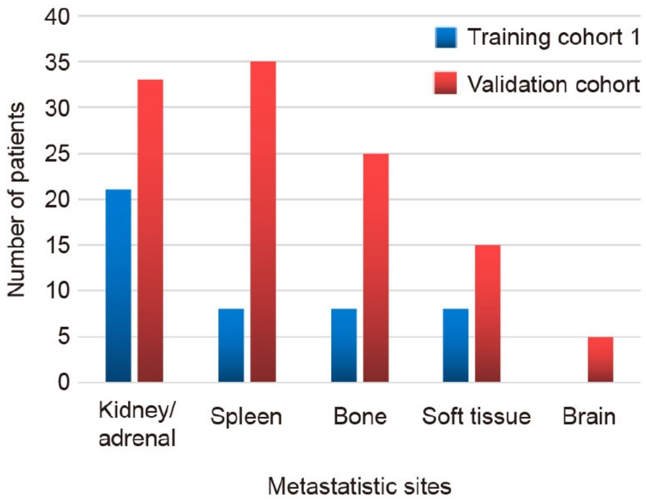

Supplementary Figure 1. The number of MPC patients with uncommon metastatic sites.

Supplementary Table 1. Clinicopathological characteristics according to metastatic sites in the validation cohort

Characteristics	Liver metastasis		P	Lung metastasis		P	Peritoneum metastasis		P
	Yes	No		Yes	No		Yes	No	
Gender									
Male	149	59	0.830	53	155	0.518	65	143	0.421
Female	91	38		37	92		35	94	
Age									
<65	120	53	0.440	38	135	0.043	52	121	0.874
≥65	120	44		52	112		48	116	
ECOG PS									
0-1	120	47	0.797	44	123	0.883	46	121	0.397
≥2	120	50		46	124		54	116	
Body mass index(kg*m-2)									
Normal weight (18.5–25)	171	69	0.983	64	176	0.979	75	165	0.319
Others	69	28		26	71		25	72	
Primary tumor location									
Head and neck	103	43	0.754	36	110	0.440	43	103	0.913
Body and tail	137	53		54	136		57	133	
CA19-9 (U/ml)									
<1000	140	69	0.013	50	159	0.165	59	150	0.409
≥1000	98	25		38	85		40	83	

Supplementary Table 2. Univariate and multivariate analysis of prognostic factors for OS in the validation cohort

	Univariate analysis			Multivariate analysis		
Characteristics	HR	95%CI	P	HR	95%CI	P
Gender						
Male	Ref					
Female	0.933	0.741-1.174	0.553			
Age						
<65	Ref					
≥65	1.150	0.921-1.435	0.216			
ECOG PS						
0	Ref			Ref		
1	1.626	1.018-2.598	0.042	1.213	0.749-1.963	0.433
2	1.844	1.163-2.924	0.009	1.387	0.861-2.233	0.179
Body mass index						
Normal weight (18.5–25)	Ref					
Others	1.035	0.811-1.321	0.783			
Primary tumor location						
Head and neck	Ref					
Body and tail	1.193	0.953-1.494	0.124			
CA19-9 (U/ml)						
<1000	Ref			Ref		
≥1000	1.311	1.042-1.651	0.021	1.250	0.988-1.581	0.063
Number of metastatic sites						
One	Ref			Ref		
Two	1.583	1.203-2.083	0.001	1.599	1.203-2.126	0.001
Three	2.266	1.645-3.121	<0.001	2.134	1.545-2.948	<0.001
More than three	2.794	1.841-4.238	<0.001	2737	1.800-4.162	<0.001
Chemotherapy						
No	Ref					
Monotherapy	0.554	0.408-0.752	<0.001	0.648	0.472-0.890	0.007
Combination therapy	0.402	0.287-0.562	<0.001	0.477	0.337-0.674	<0.001
